# Enhanced interfacial interaction between modified cellulose nanocrystals and epoxidized natural rubber via ultraviolet irradiation

**DOI:** 10.1038/s41598-022-10558-5

**Published:** 2022-04-23

**Authors:** Oranooch Somseemee, Pongdhorn Saeoui, Florian T. Schevenels, Chomsri Siriwong

**Affiliations:** 1 Materials Chemistry Research Center (MCRC-KKU), Department of Chemistry and Center of Excellence for Innovation in Chemistry (PERCH-CIC), Faculty of Science, Khon Kaen University, Khon Kaen, 40002 Thailand; 2 National Metal and Materials Technology Center (MTEC), National Science and Technology Development Agency (NSTDA), 114 Thailand Science Park, Khlong Luang, Pathum Thani 12120 Thailand; 3 Department of Chemistry, Faculty of Science, Khon Kaen University, Khon Kaen, 40002 Thailand

**Keywords:** Nanoscale materials, Mechanical properties, Polymers

## Abstract

This study aims at evaluating the reinforcement of cellulose nanocrystals (CNCs) in epoxidized natural rubber (ENR). Both CNCs and maleic anhydride-modified CNCs (M-CNCs) were prepared from Napier grass stems and characterized by various techniques (e.g., TEM, FTIR, TGA, etc.). They were incorporated into ENR latex at various loadings prior to casting, and then curing by ultraviolet (UV) irradiation. Mechanical properties of the ENR vulcanizates were finally investigated. Results revealed that the prepared CNCs had an average diameter and length of 5 nm and 428 nm, respectively. After modification, M-CNCs contained double bonds in maleate units, which could react with ENR to form covalent bonds under UV irradiation through a proposed mechanism. Regardless of the filler type, mechanical properties including hardness, modulus, and tensile strength, increased considerably with increasing filler loading. At the same filler loading, M-CNCs exhibited greater reinforcement than CNCs due to the enhanced rubber–filler interaction.

## Introduction

Polymer nanocomposites have gained much attention during the past decades due to their excellent mechanical properties. Reinforcement with various nanofillers such as silica, carbon black, carbon nanotubes, halloysite nanotubes, nanoclays, and graphene in rubbers has been widely exploited. Among these nanofillers, nanocellulose has recently received significant attention in the development of lightweight and high-performance nanocomposites due to its low specific gravity, high aspect ratio, good mechanical properties, and others including biodegradable and renewable aspects^1,2^. Because of the outstanding physical properties and sustainability of nanocellulose, there is a growing interest in using nanocellulose as a reinforcing filler in a variety of rubbers^3–5^. In general, cellulose nanoparticles are incompatible with most non-polar rubbers due to the abundance of polar functional groups on their surfaces, therefore limiting molecular scale interaction, which leads to unsatisfactory enhancement of mechanical properties^6^. In addition, the agglomeration of cellulose nanoparticles, especially at high loadings, can lead to poor filler dispersion. It has a negative impact on mechanical properties of the nanocomposite because the agglomerated cellulose nanoparticles act as stress concentration points, leading to premature failure of the composite^7^. To improve the compatibility of nanocellulose with non-polar rubbers, surface modification of nanocellulose is essential. For instance, Rosilo et al.^8^ reported the improved interaction between polybutadiene (PBD) and CNCs by modifying the CNCs’ surface with hydrocarbon chains having a terminal double bond. A UV initiator was employed together with a bifunctional dithiol cross-linker to create covalent linkages at the rubber–filler interface via a thiol–ene reaction, which was previously proposed^9^. Kanoth et al.^10^ also modified the surface of cellulose nanocrystals (CNCs) with mercapto groups and used them as a filler in natural rubber (NR). Apart from being the reinforcing filler, the modified CNCs can also act as cross-linking bridges in NR through the thiol–ene reactions leading to the improvements of filler dispersion and mechanical properties. Mariano et al.^11^ revealed enhanced interfacial interaction via hydrogen bonds (H-bonds) between the hydroxy groups on oxidized NR and those on CNCs. It has been proven that the interfacial adhesion between rubber and CNCs strongly depends on both chemical and physical interactions^12,13^. Pei et al.^14^ demonstrated the ability of CNCs to function as a chemical cross-linker in polyurethane elastomers, leading to significant improvements of both thermal and mechanical properties. Previous literature clearly shows that both filler dispersion and interaction at the nanofiller-polymer interface are the main parameters governing the properties of those nanocomposites.

This work explored the potential of using CNCs, extracted from Napier grass stems, not only as the reinforcing filler but also as the cross-linking agent in bio-based epoxidized natural rubber (ENR). To achieve a synergistic effect of reinforcement and crosslinking at the rubber–filler interface, maleic anhydride (MAH) was grafted onto the CNCs’ surface via ring-opening esterification reactions^12,15^. Although the crosslinking reaction between MAH-modified CNCs (M-CNCs) and ENR has previously been reported, a very high thermal energy was required to initiate such reaction^12^. In this study, the UV curing method was applied to induce crosslinking of the ENR nanocomposites due to its greater benefit in terms of the reduced residual chemicals in the final product, compared to the sulfur vulcanization method. The nanocomposites were prepared by mixing a variety of loadings of unmodified CNCs and M-CNCs in ENR latex, casting into thin rubber films and then, curing under UV irradiation. The mechanism for enhanced interfacial interactions between ENR and M-CNCs was also proposed. Finally, mechanical properties of the nanocomposites filled with CNCs and M-CNCs were investigated and discussed.

## Results and discussion

### Characterization of ENR

**Figure 1:**
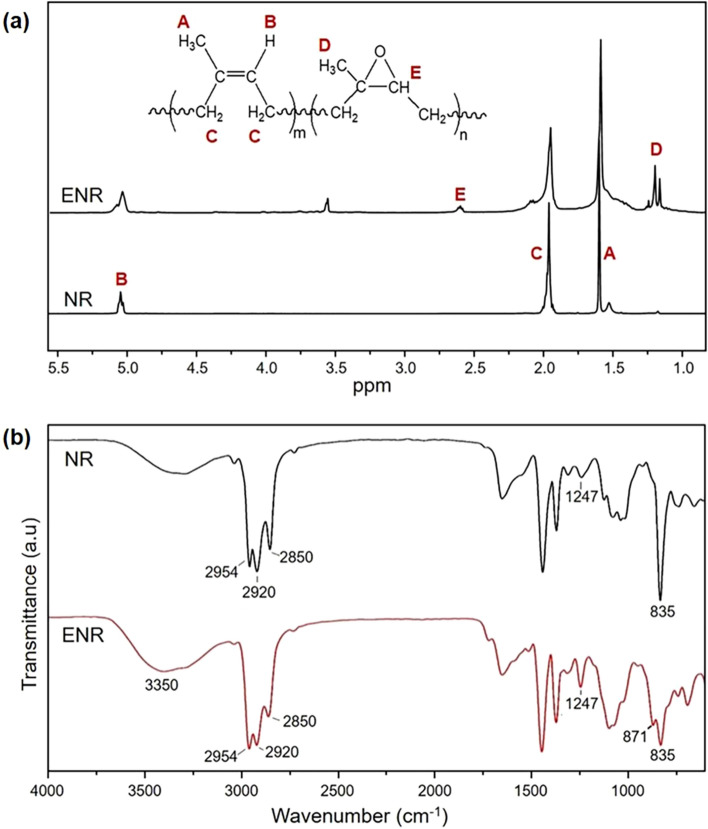
(**a**) ^1^H-NMR spectra and (**b**) FTIR spectra of NR and ENR.

In this study, ENR latex was prepared from the reaction between NR latex and performic acid. After the reaction, the obtained ENR was characterized by ^1^H-NMR and FTIR techniques. The resulting spectra are given in Fig. 1. As demonstrated in Fig. 1a, the ^1^H-NMR spectrum of NR exhibits three signals at chemical shift values of 1.6, 2.0, and 5.1 ppm, which are assigned to methyl, methylene, and unsaturated methine protons of *cis*-1,4 polyisoprene units, respectively^16^. The ^1^H-NMR spectrum of ENR shows two additional signals at chemical shift values of 1.3 and 2.7 ppm, which are attributed to methyl and methine protons of the epoxide group, respectively^17^. It is worth mentioning that there are small signals appeared at chemical shift values of 1.55, 2.05 ppm. These overlapping signals, typically observed in the ^1^H-NMR spectrum of ENR, have been reported to come from various combinations of triad sequences of epoxidized and unepoxidized isoprene units^18^. In addition, the signal at 3.6 ppm found in the ENR spectrum is attributed to the hydrogen atoms of tritron X-100, the surfactant used during the ENR synthesis^19^. The epoxide content calculated from Eq. (1) is 30% mole.

The FTIR spectra of NR and ENR are shown in Fig. 1b. The spectrum of NR is very similar to that previously reported in the literature^20^. The absorption peak at 835 cm^−1^ is assigned to C=C vibration of *cis*-1,4-polyisoprene. Moreover, the absorption peaks at 2850 cm^−1^ and 2920 cm^−1^ are attributed to asymmetric and symmetric stretching vibrations of CH_2_ (methylene group) in NR, respectively. The NR spectrum also shows a strong peak at around 2954 cm^−1^, corresponding to C–H vibration of methyl groups in NR^21^. For ENR, the two absorption peaks observed at 871 cm^−1^ and 1247 cm^−1^ belong to asymmetric and symmetric stretching vibrations of the C–O–C of the epoxide groups in ENR chains, respectively^22^. The intensity increment of the broad absorption peak at 3350 cm^−1^ is likely due to the presence of additional –OH groups obtained from epoxide ring-openings that may take place, especially at high epoxide contents^23^.

### Characterization of CNCs and M-CNCs

**Figure 2:**
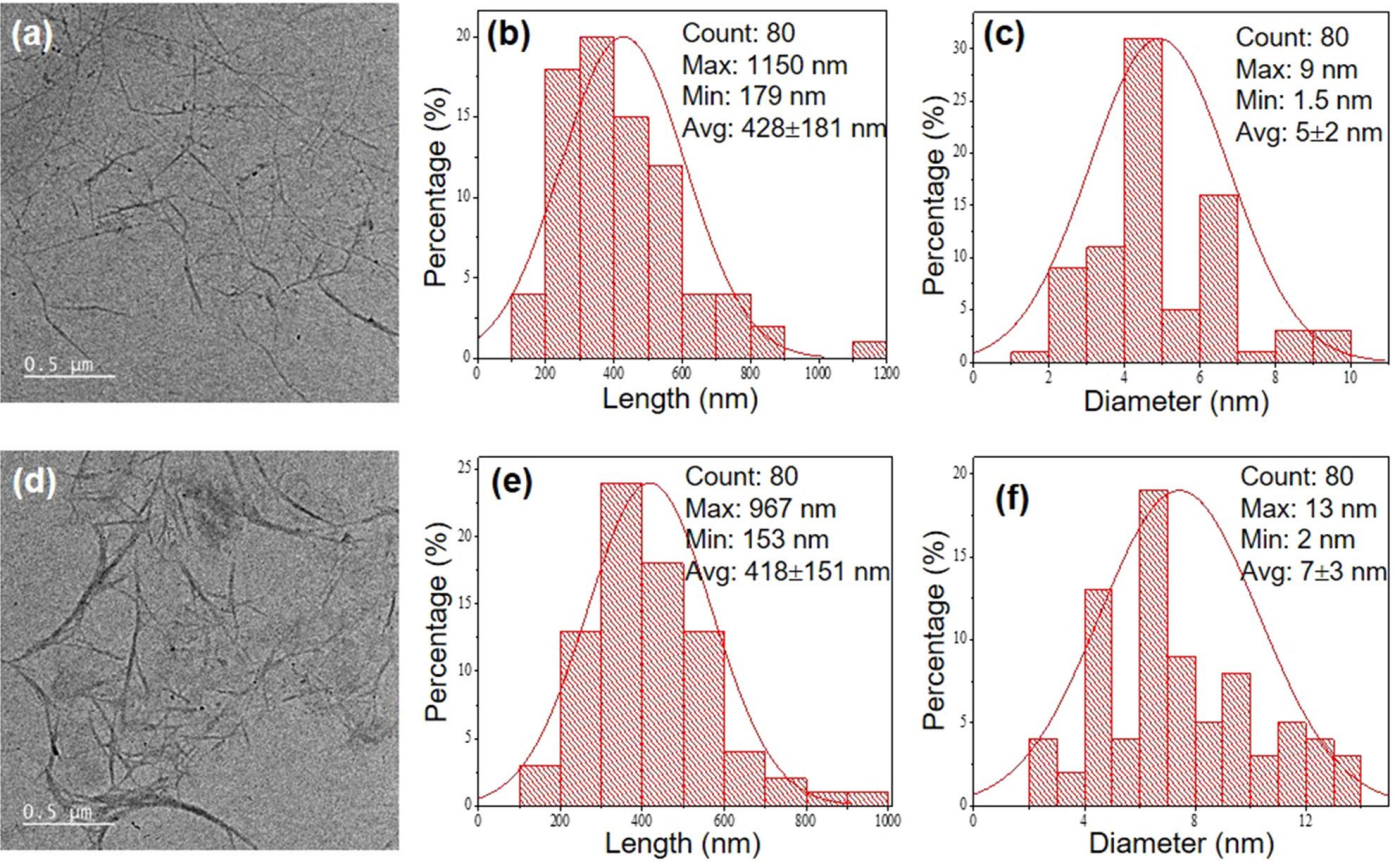
TEM images and size distributions of (**a**, **b**, and **c**) CNCs and (**d**, **e**, and **f**) M-CNCs.

In this study, CNCs were extracted from Napier grass stems through alkali-steam explosion, bleaching, and acid hydrolysis treatments. The CNC extraction process is schematically presented in Fig. S1 in the Supplementary Information. The yield of CNC extraction with respect to the initial amount of the dried Napier grass stem was approximately 24%. TEM images in association with the length and diameter distributions of CNCs and M-CNCs are illustrated in Fig. 2. Both CNCs and M-CNCs have a rod-like shape with a very high aspect ratio. They also exhibit good dispersion in aqueous suspension with relatively large distributions in length and diameter. The good dispersion is thought to arise from the reaction between the sulfuric acid and the hydroxyl groups on the cellulose’s surface via an esterification during the synthesis, allowing the grafting of anionic sulfate ester groups on the surfaces of CNCs and M-CNCs. The presence of sulfate groups significantly increases the negative charge density on the cellulose’s surface leading to the good dispersion of CNCs and M-CNCs in aqueous suspension. The length of CNCs varies from 179 to 1150 nm, with an average value of 428 nm. The MAH modification does not significantly affect the length of the CNCs, i.e., the average length of the M-CNCs is around 418 nm. However, the average diameter of the CNCs slightly increases from 5 to 7 nm after the MAH modification, which may be explained by the attachment of the MAH on the CNCs’ surface. The average values of diameter and length of the CNCs are comparable to those previously reported in the literature^1,12^.

**Figure 3:**
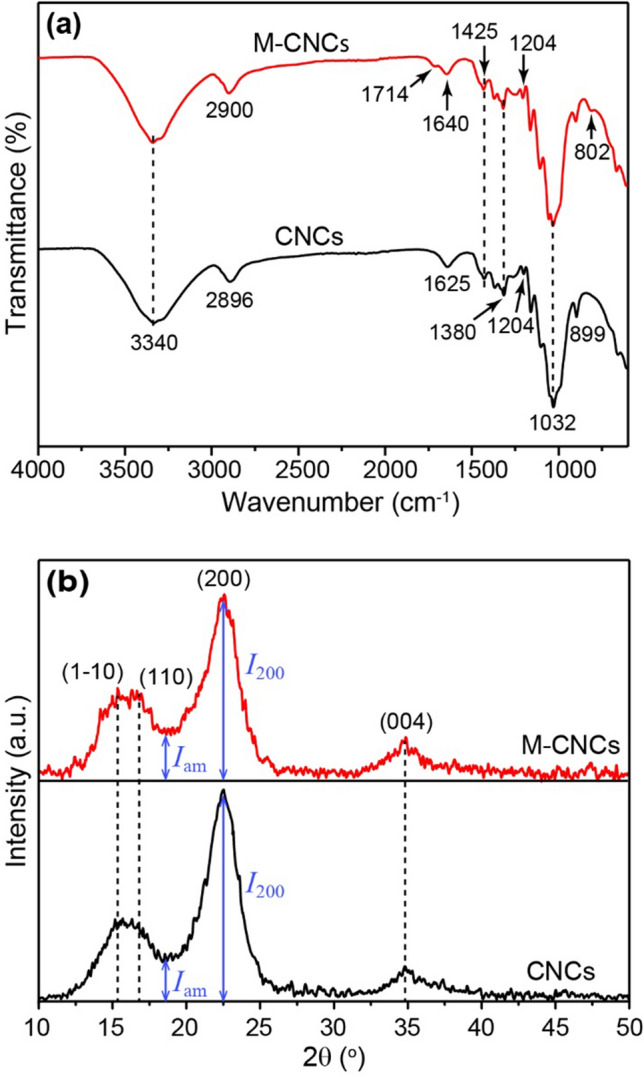
(**a**) FTIR spectra and (**b**) XRD patterns of CNCs and M-CNCs.

Functional groups of CNCs and M-CNCs were determined by FTIR, and the results are given in Fig. 3a. The CNC spectrum possesses three main characteristic peaks at 1032 cm^−1^, 3340 cm^−1^, and 2900 cm^−1^, corresponding to the stretching vibrations of C–O–C, O–H, and C–H bonds, respectively. The broad peak at 3340 cm^−1^ also includes inter- and intra-molecular H-bond vibrations in cellulose^24^. The peak at 1625 cm^−1^ is attributed to the O–H bending vibration of water molecules adsorbed on the CNCs’ surface. The peak located at 1425 cm^−1^ is attributed to CH_2_ intertwined in the cellulosic material. The peak observed at 899 cm^−1^ is reported to be associated with the cellulosic *β*-glycosidic linkages^25^. For M-CNCs, additional peaks are observed at wavenumbers of 1714 cm^−1^, 1380 cm^−1^, and 802 cm^−1^. The peak at 1714 cm^−1^ is attributed to the C=O stretching vibration of carboxylic groups, while the one at 1380 cm^−1^ belongs to the C–O–H deformation vibration. The presence of these two peaks clearly confirms the existence of carboxylic groups on the M-CNCs’ surface. The small peaks at 1640 cm^−1^ and 802 cm^−1^ are attributed to the stretching and bending vibrations of C=C bond, indicating the presence of double bonds in M-CNCs. FTIR results of the M-CNC sample are in good agreement with the proposed chemical structure of M-CNCs shown in Fig. S2, in which MAH reacts with hydroxy groups present in cellulose via esterification^26^. In addition, a small peak can be found at 1204 cm^−1^ in both CNCs’ and M-CNCs’ spectra. This peak is attributed to S=O vibration from the sulfate groups attached on the cellulose surface during the acid hydrolysis treatment^27^. The absorption peak of sulfate groups is more noticeable in the M-CNCs’ spectrum, indicating a greater content of sulfate groups after modification with MAH in the presence of strong sulfuric acid (H_2_SO_4_).

Further evidence of the grafting success of MAH onto the CNCs’ structure can be seen from the quantitative analysis of the carboxylic acid content in the M-CNC sample via the acid–base titration method. In this study, the number of carboxylic groups attached on the M-CNCs’ surface is 6.12 mmol/g.

Figure 3b displays the XRD patterns of CNCs and M-CNCs. The diffraction pattern of CNCs shows the 15.3°, 16.3°, 22.5° and 34.8° 2θ reflections assigned to the (1–10), (110), (200) and (004) crystallographic planes, respectively^28^. A similar diffraction pattern is obtained for M-CNCs. The peak intensity, however, tends to decrease after modification with MAH, indicating the reduction of crystallinity, i.e., the *CI* value declines from 82.2% (CNCs) to 78.1% (M-CNCs). The results indicate that the crystal structure of CNCs is partially destroyed after modification with MAH. According to the literature^12,29^, the reaction between any reagent and cellulose generally occurs either in the amorphous regions or at the edges of the crystalline regions of cellulose. The reagent initially reacts with the chain ends on the surface of crystallites as it cannot diffuse into the crystalline region, giving rise to the opening of some of the hydrogen-bonded cellulose chains, resulting in a conversion of some crystalline cellulose to amorphous cellulose. The reagent can then penetrate further into this newly produced amorphous section and react with the crystalline cellulose, resulting in the greater transformation of crystalline cellulose to amorphous cellulose.

**Figure 4:**
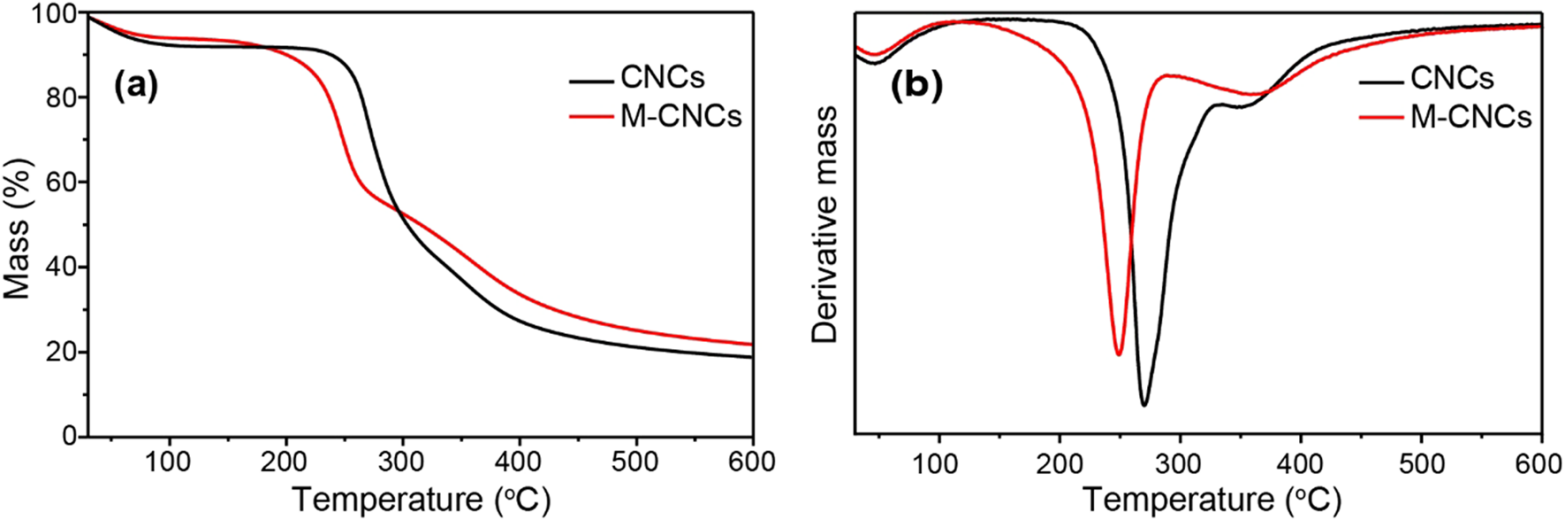
(**a**) TGA curves and (**b**) DTG curves of CNCs and M-CNCs.

TGA and DTG curves of CNCs and M-CNCs are shown in Fig. 4a and b. The initial mass loss (~ 7–8%) in the range of 50–100 °C found in both samples, corresponding to the removal of adsorbed moisture on the cellulose surface^30^. The mass loss found at higher temperatures is associated with the decomposition of cellulose. It has been previously reported that the cellulose decomposition begins with the cleavage of glycosidic linkages, followed by the dehydration, and, finally, the disintegration of charred residues into gaseous products^11,30^. As can be seen, TGA curves of the CNC and M-CNC samples show two decomposition steps as evidenced by the two dTG peaks at approximately 270 °C and 350 °C for the CNC sample, and 250 °C and 360 °C for the M-CNC sample. The two-step decomposition of sulfuric acid-hydrolyzed CNCs has previously been reported^31,32^. The first step, found at temperatures between 200 and 300 °C, involves the decomposition of most accessible amorphous regions or external chains that are also highly sulfated. The second step, taking place at higher temperatures, is ascribed to the decomposition of less accessible interior crystalline regions, which are comparatively less sulfated. The thermal stability of sulfuric acid-hydrolyzed CNCs is therefore greatly dependent on the hydrolysis conditions^31,33^. It has been reported that the sulfate groups lower thermal stability of CNCs^34^. Therefore, the decomposition temperature at the first step of M-CNCs is lower than that of CNCs because M-CNCs possess higher amount of sulfate groups on their surface. However, it appears that the decomposition temperature at the second step of M-CNCs is slightly higher than that of CNCs. The existence of carboxylic groups on the M-CNCs’ surface may account for this observation, as it has been reported that, during the decomposition of the outer surfaces, carboxylic groups can promote caramelization at the sample surface, effectively coating the material core, and therefore slowing down the decomposition^32,35^. At the end of the pyrolysis process, the numbers of charred residues are approximately 20% and 23% for the CNC and M-CNC samples, respectively.

### Properties of the UV-cured nanocomposites

**Figure 5:**
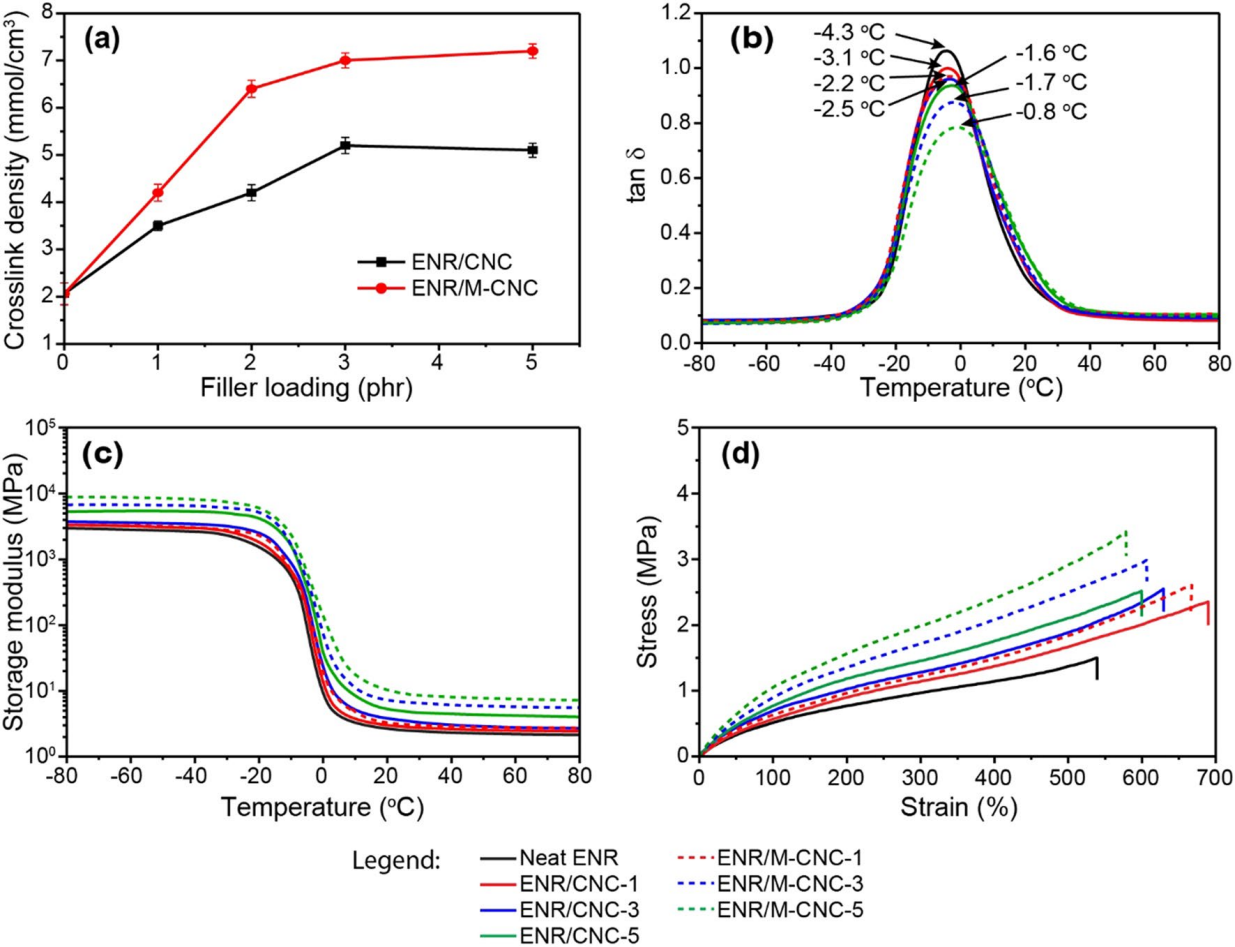
(**a**) Crosslink density, (**b**) tan δ versus temperature curves, (**c**) storage modulus (E′) versus temperature curves (**d**) tensile stress–strain curves of the nanocomposites.

Crosslink densities of the UV-cured nanocomposites calculated from Flory–Rehner equation (Eq. 4) are displayed in Fig. 5a.

**Figure 6:**
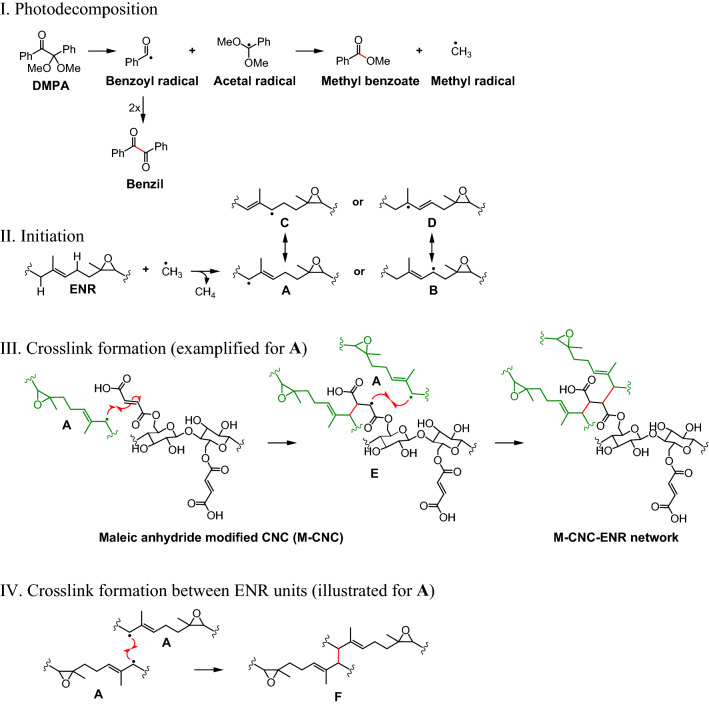
Proposed crosslink mechanism under UV irradiation of the ENR/M-CNC nanocomposites.

Regardless of the filler type, crosslink density tends to increase rapidly with the increment of filler loading up to 3 phr before leveling off. The initial increase in crosslink density may be attributed to strong rubber–filler interactions, as tightly-bound rubber could resist solvent swelling to a greater extent than the rubber matrix. Similar observations have been reported elsewhere^3,36^. At higher filler loading (5 phr), the fillers tend to aggregate, leading to insignificant increases in bound rubber content and, hence, crosslink density. At any given filler loading, M-CNCs provide higher crosslink density than CNCs. The results are not beyond expectation because it has been reported that the carboxylic groups of M-CNCs can react with ENR at high temperature (180 °C) and form covalent linkages via transesterification of *β*-hydroxy ester linkages, leading to additional crosslinks^12,37^. Consequently, M-CNCs not only act as a filler, but also function as a crosslink bridge between rubber molecules. However, in this study, the crosslink mechanism at the rubber–filler interface is different because the reactions are triggered by UV irradiation in the presence of a photo-initiator, namely DMPA. The crosslink mechanism is proposed in Fig. 6.

A photo-initiated free radical reaction typically has four steps, i.e., photo-decomposition, initiation, propagation, and termination^38^. In this case, DMPA is activated by UV irradiation and decomposes into benzoyl and acetal radicals (Fig. 6(I)). The latter undergoes fragmentation, yielding methyl benzoate and methyl radicals that act as initiating radicals for the forthcoming reactions. Initiation (Fig. 6(II)) occurs at allylic positions of the ENR, after hydrogen abstraction by either methyl or benzoyl radicals, generating different forms of allylic radical (**A**–**D**). Any of these radicals can react with double bonds in maleate units of M-CNCs through radical conjugate addition, to form the first linkage and a new radical (**E**) that can further react with the allylic radical of another ENR molecule, resulting in the complete formation of crosslink as shown in Fig. 6(III). Additionally, two allylic radicals of ENR can react with each other to form a network in ENR itself (Fig. 6(IV)). At the end of the curing process, a complex network with different types of linkages is therefore created in the ENR/M-CNC nanocomposites. It should be noted that other side reactions may also occur, particularly at the rubber surface where the presence of oxygen is inevitable. In this case, the allylic radicals can react with oxygen and form peroxyl radicals (ROO˙) and finally turn into hydroperoxides (ROOH), which can further break into aldehydes or ketones, widely known as oxidative chain scission. However, no sign of surface degradation was observed under the employed irradiation conditions.

**Figure 7:**
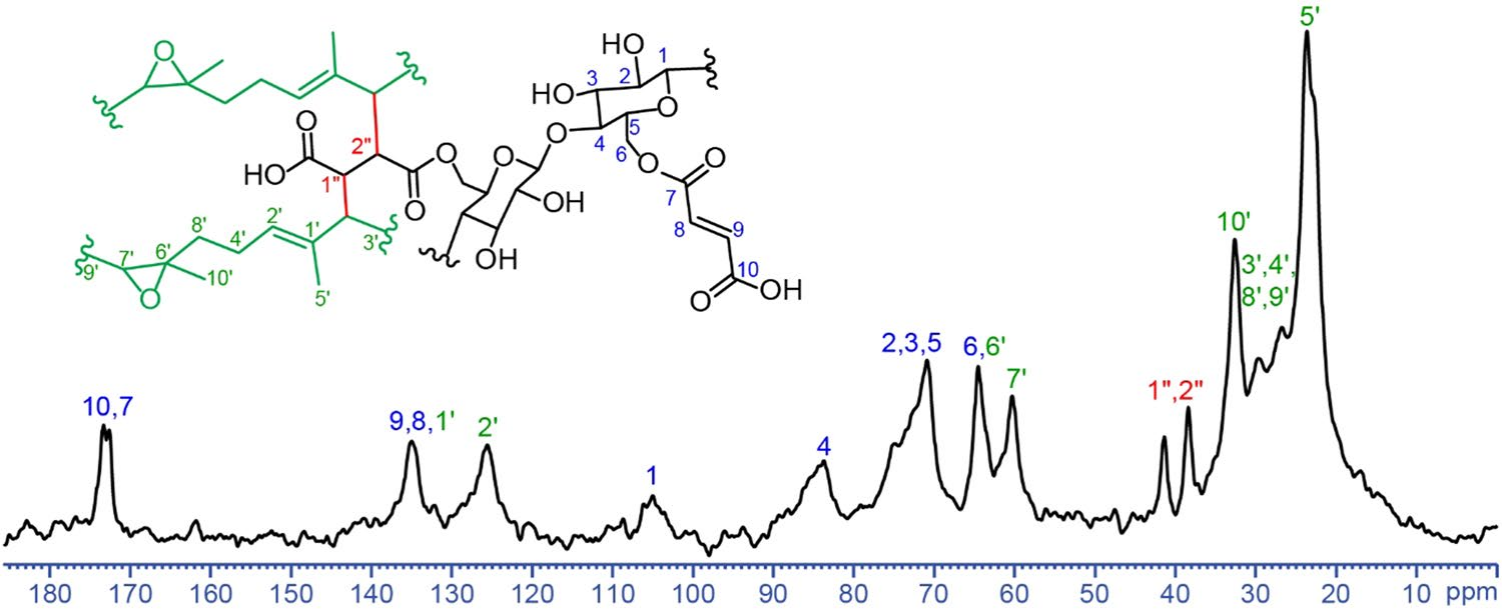
^13^C cross polarization-magic angle spinning (CP-MAS) NMR spectrum of the ENR/M-CNC composite.

To prove the proposed reaction, the ENR/M-CNC nanocomposite was characterized by ^13^C-NMR technique and the NMR spectrum is displayed in Fig. 7. The signals found between 64 and 107 ppm are attributed to the carbon atoms (C1–C6) of cellulose fragment, while those appearing at 172–173 ppm and around 135 ppm belong to carbon atoms of C=O (C7 and C10) and C=C (C8 and C9) groups, respectively, indicating the successful grafting on the CNCs’ surface after the MAH modification. For the ENR fragment, the signals at 23 and 33 ppm represent C5′ and C10′ of the methyl carbons. The signals of C3′, C4′, C8′ and C9′ appear within the aliphatic region (26 to 32 ppm). The signals at 60 and 64 ppm represent C6′ and C7′ of the oxirane. The signals at 125 and 135 ppm belong to C2′ and C1′ of the alkene in the isoprene units^39^. When compared with the NMR spectra of ENR and M-CNC respectively disclosed in Refs.^39,40^, it becomes clear that the NMR spectrum of the ENR/M-CNC composite retains features of both ENR and M-CNC spectra. However, there are two new signals appearing at 38 and 41 ppm, which belong to methine carbons (C1″ and C2″) adjacent to carbonyl groups, indicating the successful crosslinks.

Unlike CNCs, M-CNCs can induce chemical bonds at the rubber–filler interface, making it more difficult for solvent to diffuse into the specimens. The ENR/M-CNC nanocomposites therefore show higher crosslink density than the ENR/CNC nanocomposites. The increase in crosslink density may also reduce the rubber chain mobility, which could be indirectly characterized by the measurement of glass transition temperature (Tg)^41^. The relationship between tan δ and temperature of the ENR nanocomposites is displayed in Fig. 5b, in which the temperature at maximum tan δ is generally used to represent Tg of the composites. Clearly, Tg tends to increase with increasing filler loading for both fillers. Again, at the same filler loading, the ENR/M-CNC nanocomposites exhibit higher Tg than the ENR/CNC nanocomposites, indicating the greater restriction of chain movement during the transition as a result of the higher crosslink density, as previously mentioned. From literature, the area of the tan δ_max_ peak can be used to reflect the number of rubber molecules participating in the transition^42^. From Fig. 5b, the peak area declines continuously with increasing filler loading due to the dilution effect. At the same filler loading, a lower peak area is observed in the ENR/M-CNC nanocomposites, possibly due to greater rubber–filler interactions. In addition to the tan δ results, the results of storage modulus (E′) as a function of temperature are also given in Fig. 5c. In the glassy region, molecular movement is completely restricted and, hence, extremely high values of E′ are observed (3000–9000 MPa). Rapid change of E′ is found at the transition temperature. In the rubbery region where a greater molecular movement is permitted, the E′ value considerably drops to below 10 MPa depending on the type and the content of filler. At room temperature (25 °C), E′ increases with increasing filler loading, which could be explained by the increased crosslink density (see Fig. 5a) and the dilution effect, leading to greater resistance to deformation of the nanocomposites^43^. At the same filler loading, M-CNCs impart higher E′ than CNCs because of their higher rubber–filler interactions and, thus, crosslink density. The mechanical properties of the ENR/CNC and ENR/M-CNC nanocomposites were also determined, and the results summarized in Table 1.

**Table 1:** Mechanical properties of the ENR nanocomposites.

Composite	Loading (phr)	Hardness (Sh A)	M100 (MPa)	TS (MPa)	Elongation at break (%)
Neat ENR	0	34.6 ± 1.2	0.49 ± 0.01	1.5 ± 0.4	545 ± 30
ENR/CNC	1	37.1 ± 0.7	0.55 ± 0.01	2.4 ± 0.1	690 ± 20
	2	38.2 ± 0.5	0.62 ± 0.04	2.5 ± 0.1	650 ± 15
	3	39.8 ± 0.3	0.71 ± 0.05	2.6 ± 0.1	630 ± 23
	5	40.9 ± 0.6	0.80 ± 0.05	2.5 ± 0.1	600 ± 12
ENR/M-CNC	1	38.6 ± 0.5	0.64 ± 0.01	2.6 ± 0.1	668 ± 64
	2	40.7 ± 0.4	0.74 ± 0.01	2.7 ± 0.1	630 ± 15
	3	43.7 ± 1.5	0.90 ± 0.04	3.0 ± 0.1	610 ± 33
	5	45.9 ± 0.3	1.01 ± 0.16	3.4 ± 0.1	580 ± 41

As hardness is a material property indicating the deformation resistance at very low strains of a material, hardness and modulus are closely related. Examples of engineering stress–strain curves of the unfilled ENR and the ENR nanocomposites filled with 1, 3 and 5 phr of fillers are also given in Fig. 5d. Both hardness and tensile modulus (stress) at 100% elongation (M_100_) follow the same trend as the dynamic storage modulus (E′), i.e., they increase with increasing filler loading, and M-CNCs give higher values than CNCs. The same explanation applies. Similar results have been revealed by Kanoth et al.^10^ for the CNC-filled natural rubber nanocomposites, in which hardness and modulus increase after the addition of CNCs. Without filler, tensile strength and elongation at break are 1.49 MPa and 545%, respectively. Tensile strength substantially increases in the presence of 1 phr of CNCs and M-CNCs. Further increase in filler loading also results in a continuous increase in tensile strength of the nanocomposites, which is particularly obvious in the M-CNC filled system, indicating the high reinforcing ability of both CNCs and M-CNCs. As can be expected, M-CNCs provide a greater rubber–filler interaction via covalent bonds, promoting the efficient transfer of stress from rubber to filler^12,44^. Therefore, M-CNCs show significantly higher tensile strength than CNCs. Similar observation has been reported by Cao et al. who found the significant increase in tensile strength after the tunicate CNCs were modified by MAH^12^. They also found that, when MAH-modified tunicate cellulose nanocrystals (M-t-CNCs) were added into ENR at 5 phr, the tensile strength of the obtained nanocomposite was approximately 3.5 MPa which is comparable to the tensile strength of the nanocomposite filled with 5 phr of M-CNCs in this work. Despite the dilution effect and the increase in crosslink density, elongation at break slightly increases when 1 phr of CNCs or M-CNCs is added. This unexpected observation has also been reported elsewhere. It was proposed to arise from the dissociation of hydrogen bonds between nanocellulose-nanocellulose, nanocellulose-ENR, and ENR-ENR in association with the partial orientation of the CNCs during stretching, which help to dissipate energy and improve the extensibility of the nanocomposites^10,12,36^. Further addition of the filler above 1 phr, however, results in the continuous reduction of elongation at break, possibly due to the dominant dilution effect accompanied by the increased crosslink density. This result is easily understandable because both CNCs and M-CNCs are much stiffer and less extendable than ENR. Therefore, increasing filler loading causes the reduction of ENR proportion in the specimens, resulting in a decrease in strain at failure. At any given filler loading, the ENR/M-CNC nanocomposites exhibit slightly lower elongation at break than the ENR/CNC nanocomposites. The increased crosslink density arising from the chemical interaction between ENR and double bonds in maleate units of the M-CNCs under UV irradiation may be used to explain this finding.

**Figure 8:**
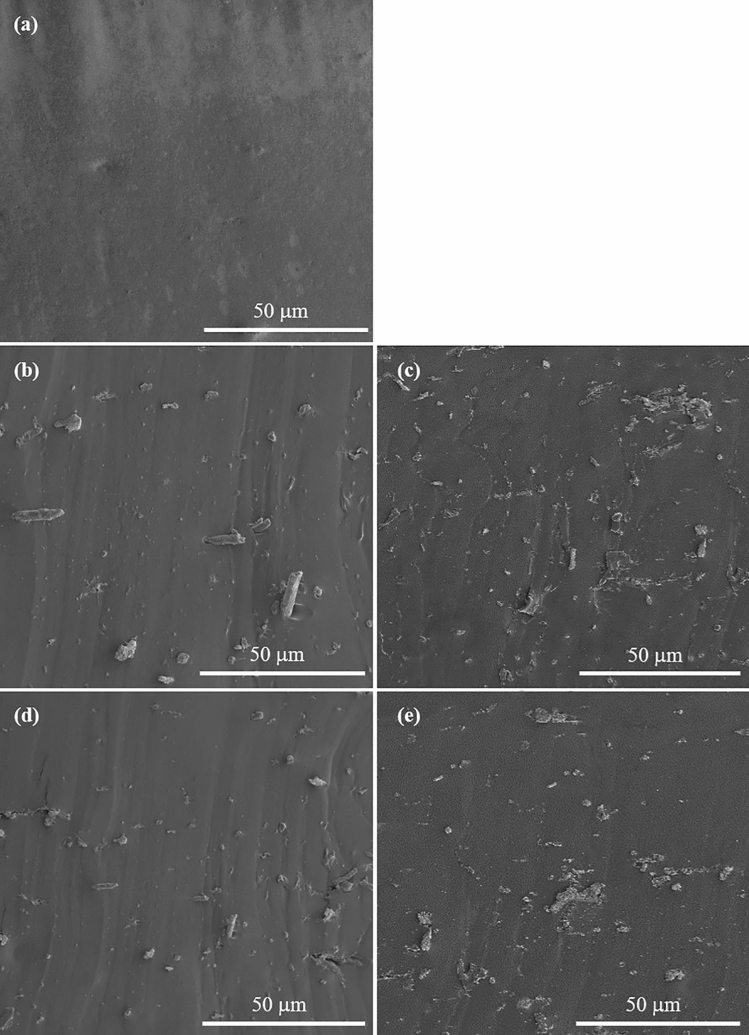
SEM images of the tensile fractured surfaces of: (**a**) Neat ENR, (**b**) ENR/CNC at 3 phr, (**c**) ENR/CNC at 5 phr, (**d**) ENR/M-CNC at 3 phr, and (**e**) ENR/M-CNC at 5 phr.

Figure 8 shows the examples of SEM micrographs of the nanocomposites filled with different loadings of CNCs and M-CNCs. Clearly, filler agglomeration is observed in both CNC-filled and M-CNC-filled systems, particularly pronounced at high filler loading. A slight improvement of filler dispersion is observed after the MAH modification. This can be explained by the reduced hydrophilicity of M-CNCs due to the presence of long hydrocarbon chains after the grafting that inhibits the tendency to form agglomerates^45^.

## Conclusions

In this work, cellulose nanocrystals (CNCs) have been successfully prepared from Napier grass stems (*Pennisetum purpureum*), with 24% yield, using the following treatments, i.e., alkali-steam explosion, bleaching and acid hydrolysis. Various characterizations show that the prepared CNCs have a rod-like shape with an average diameter, length, and aspect ratio of 5 nm, 418 nm, and 84, respectively. Due to its nanoscale size, the addition of untreated CNCs into ENR results in significant increases in modulus and hardness with the sacrifice of elongation at break. Tensile strength also increases with increasing CNC loading up to 3 phr before leveling off. Rubber–filler interaction is mainly governed by Van der Waals forces and hydrogen bonds in this system. After grafting with MAH, both diameter and length of CNCs do not significantly change. When incorporated into ENR, M-CNCs reinforce rubber in a similar manner to CNCs, i.e., the changes of mechanical properties with increasing filler loading follow the same trends. However, the presence of double bonds in maleate units of M-CNCs permits the formation of covalent bonds at the rubber–filler interface through free radical reactions triggered by UV irradiation, leading to an additional crosslink network. The enhanced interfacial interactions lead to a significant improvement of mechanical properties. For instance, when loaded at 3 phr, M-CNCs show 15.0% and 26.7% increases in tensile strength and modulus, respectively, compared to CNCs.

## Materials and methods

High ammonia natural rubber latex (HA-NR) with a dry rubber content (DRC) of 60% was supplied by Thai rubber latex group PCL., Samut Prakan, Thailand. Napier grass stems were obtained from Bureau of Animal Nutrition Development, Khon Kaen, Thailand. The collection and experimental research on Napier grass comply with Plant Variety Protection Act, Thailand (1999). Sulfuric acid (H_2_SO_4_), hydrogen peroxide (H_2_O_2_), *N*,*N*-dimethylformamide (DMF), acetic acid (CH_3_CO_2_H), and ethanol (C_2_H_5_OH) were purchased from QRëC, New Zealand. Maleic anhydride (C_4_H_2_O_3_) was obtained from Merck, Darmstadt, Germany. Triton-X 100, formic acid (HCO_2_H), sodium hydroxide (NaOH), and toluene (C_6_H_5_CH_3_) were supplied by Loba Chemie PVT. Ltd., Mumbai, India. 2,2-Dimethoxy-2-phenylacetophenone (DMPA) was purchased from Sigma-Aldrich, Missouri, USA. Sodium chlorite (NaClO_2_) was purchased from Ajax Finechem, Auckland, New Zealand.

### Synthesis of epoxidized natural rubber (ENR)

ENR latex was prepared by mixing the diluted high ammonia natural rubber latex (20% DRC) with 3.3 phr of triton-X 100 (0.70 g) as a non-ionic surfactant. The mixture was stabilized at room temperature for 24 h. The mixture temperature was raised to 60 °C before adding formic acid (molar ratio of HCO_2_H/isoprene = 0.5 mol/mol) and stirred for 10 min under nitrogen gas. Then, hydrogen peroxide (molar ratio of H_2_O_2_/isoprene = 1.5 mol/mol) was added dropwise. The mixture was then stirred at a speed of 500 rpm for 6 h. The obtained latex was subsequently characterized by Fourier transform infrared spectroscopy (FTIR, Bruker Tensor 27, Ettlingen, Germany) and Proton Nuclear Magnetic Resonance spectroscopy (^1^H-NMR, NMR-400 MHz, Avance Neo, Bruker, Switzerland). The epoxide content of ENR was determined from ^1^H-NMR results using Eq. (1)^46^.

1 $$mol\% \;epoxide = \frac{{I_{2.7} }}{{I_{2.7} + I_{5.1} }} \times 100$$

where *I*_2.7_ and *I*_5.1_ are the integrations of the resonances appearing at chemical shifts of 2.7 and 5.1 ppm, which correspond to the epoxy methine of epoxidized-*cis*-1,4-isoprene units and to the olefinic methine of unsaturated *cis*-1,4-isoprene units, respectively.

### Extraction of CNCs from Napier grass stems

The CNCs were isolated from Napier grass stems using the procedures described in the literature^2,47^. The CNC extraction process is schematically presented in Fig. S1. Napier grass stems were firstly cut into small pieces (< 1 mm) and dried overnight at 60 °C. The dried sample (1–1.5 g) was treated by 5 wt% NaOH solution (10 mL) at 70 °C for 2 h under a continuous stirring, and then autoclaved in a pressure steam sterilizer (ALL AMERICAN model no. 75X, Hillsville, Virginia, USA) for 3 h at a pressure and temperature of 110 kPa and 121 °C, respectively. The sample was then filtered and thoroughly washed with hot distilled water (70 °C) and dried overnight in an oven at 80 °C. Delignification was performed by treating the sample with a bleaching agent, a mixture of acetate buffer and sodium chlorite solution (1:1 v/v), at 80 °C for 2 h. The bleached sample (3.5 g) was later mixed with 64 wt% sulfuric acid (70 mL) and stirred vigorously at 50 °C for 50 min. The resultant fibers were thoroughly washed to ensure the complete removal of sulfuric acid and followed by dialysis in distilled water to neutralize the pH. The CNC suspension was finally subjected to a vigorous stirring using a homogenizer (HG-15A, Daihan Scientific, Korea) prior to being freeze-dried to obtain the dried CNC sample.

### Modification of CNCs with maleic anhydride

Firstly, 1 g of the CNC sample was added into 20 mL of DMF. After vigorous stirring to ensure good dispersion, 0.08 mol of MAH were added and kept stirring until MAH dissolved completely. Next, 1 mL of catalyst (98% H_2_SO_4_) was slowly added under stirring. The mixture was then refluxed at 120 °C for 10 h to allow esterification to occur, washed with deionized water and followed by ethanol several times using a centrifugation method (at 6,000 rpm for 10 min) to ensure complete removal of the unreacted MAH. Finally, the mixture was freeze-dried to give a white powder, denoted as M-CNC sample. The chemical reaction is represented in Fig. S2.

### Characterization of CNC and M-CNC samples

Morphologies of CNCs and M-CNCs were examined under a transmission electron microscope (TEM, TECNAI G^2^ 20STwin, FEI Company, Oregon, USA). A droplet of the diluted CNC and M-CNC suspensions was deposited onto a carbon-coated copper grid and allowed to dry at room temperature prior to the examination. ImageJ software (US National Institutes of Health, Bethesda, Maryland, USA) was used to evaluate the average values of diameter and length. Surface functional groups were examined by Fourier transform infrared (FTIR) spectrophotometer equipped with an attenuated total reflectance (ATR) facility (Bruker Tensor 27, Ettlingen, Germany). The dried samples were placed in a vacuum oven at 60 °C for 24 h to remove adsorbed moisture prior to the analysis. Thermogravimetric analyzer (TGA, STA7200 Hitachi, Tokyo, Japan) was used to study thermal degradation behaviors of the dried samples. The test was carried out under nitrogen gas from 30 to 600 °C at a heating rate of 10 °C/min. The degree of carboxylic groups on the M-CNCs’ surface was determined by back titration method^48^. To do so, 0.1000 g of the M-CNC sample was mixed with 100 mL of 0.010 M NaOH solution. After stirring for 2 h, the suspension was filtered and 25 mL of the filtrate was titrated with a standardized 0.010 M HCl solution. The concentration of carboxylic groups [C_COOH_ (mmol/g)] was calculated by using Eq. (2).

2 $$C_{{{\text{COOH}}}} = \frac{{(C_{{{\text{NaOH}}}} \times V_{{{\text{NaOH}}}} - 4 \times C_{{{\text{HCl}}}} \times V_{{{\text{HCl}}}} )}}{{M_{{\text{M - CNC}}} }} \times 1000$$

*C*_NaOH_ and *C*_HCl_ are the concentrations (M) of the NaOH and HCl solutions, respectively. *V*_NaOH_ is the volume of the NaOH solution employed during the extraction (0.1 L) while *V*_HCl_ is the volume of the HCl solution at the end point of the titration. *M*_M-CNC_ is the mass of the M-CNC sample used in this experiment (0.1 g).

Crystalline structures of the samples were investigated by X-ray diffraction (XRD, Malvern Panalytical Co., Ltd., Royston, UK) using a Cu-Kα radiation with wavelength of 0.15406 nm. The crystallinity index (*CI*) was estimated using the empirical method following Eq. (3)^49^.

3 $$CI{\kern 1pt} (\% ) = \frac{{(I_{200} - I_{{{\text{am}}}} )}}{{I_{200} }} \times 100$$

where *I*_200_ is the maximum intensity of the (200) reflection at 22.5° 2θ and *I*_am_ is the intensity diffraction of the amorphous band (18.5° 2θ).

### Preparation of ENR/CNC and ENR/M-CNC nanocomposites

Various loadings of the CNC and M-CNC samples (0, 1, 2, 3, and 5 phr) were added into ENR latex and vigorously stirred for 8 h at room temperature to achieve good filler dispersion. A co-agent solution was prepared by dissolving 0.72 g of DMPA in 0.70 g of triton-X 100 in an amber glass bottle under stirring until DMPA was completely dissolved. The co-agent solution was then added into the mixtures (the total quantity of DMPA was kept constant at 0.72 g), and mixed by a magnetic stirrer for 1 h in the dark. The mixtures were later poured into Petri dishes, gradually dried at room temperature for 24 h, and then placed in an oven at 40 °C for 72 h until the nanocomposite films were completely dried. Finally, the nanocomposite films with a thickness of about 1.2 mm were irradiated for 45 min in a UV light box equipped with a 400 W metal halide lamp (UV lamps, model RUV 695 BC, Thai Inter Lamp Co., Ltd., Thailand) having a radiation wavelength (λ) in the range of 200–280 nm and the intensity of 225 mW/cm^2^. The distance between the nanocomposite films and the focal point of the UV lamp was 10 cm. Figure S3 schematically illustrates the preparation of UV-cured nanocomposite films, together with the appearance of the films before and after the UV irradiation.

### Characterization of ENR nanocomposite films

Solid-state ^13^C NMR was used to confirm the interaction between ENR and M-CNC. The experiment was performed on a Bruker Avance III NMR spectrometer (NMR-400 MHz, Bruker, Switzerland). Crosslink density of the ENR nanocomposites was determined by equilibrium swelling technique. Three specimens with the dimensions of 2 cm × 2 cm × 1.2 mm were prepared, weighed, and soaked in toluene at room temperature for 120 h to ensure equilibrium swelling. The swollen specimens were then removed from the solvent, wiped with filter paper and weighed immediately. The specimens were subsequently dried in an oven at 60 °C for 24 h before measuring the final weight. Flory–Rehner equation (Eq. 4) was employed to calculate the approximate values of crosslink density (*ν*_*e*_) of the samples.

4 $$\nu_{e} = - \frac{{\ln \left( {1 - V_{r} } \right) + V_{r} + \chi V_{r}^{2} }}{{V_{1} \left( {V_{r}^{{{1 \mathord{\left/ {\vphantom {1 3}} \right. \kern-\nulldelimiterspace} 3}}} - {{V_{r} } \mathord{\left/ {\vphantom {{V_{r} } 2}} \right. \kern-\nulldelimiterspace} 2}} \right)}}$$

and

$$V_{r} = \frac{{(m_{2} \times m_{r} ){/}\rho_{r} }}{{(m_{2} \times m_{r} ){/}\rho_{r} + (m_{{^{2} }} \times m_{f} ){/}\rho_{f} + (m_{1} - m_{2} ){/}\rho_{s} }}$$

where *V*_*r*_ is the volume fraction of rubber in the swollen specimen; *V*_1_ and *χ* are the molar volume of toluene (106.2 cm^3^/mol) and the Flory–Huggins interaction parameter between rubber and solvent (0.34 for ENR-toluene system), respectively; *m*_*1*_ and m_2_ represents the weights of samples in the swollen and un-swollen states; *ρ*_*r*_, *ρ*_*s*_ and *ρ*_*f*_ are densities of the rubber, solvent and filler, respectively. The parameters of *m*_*r*_ and *m*_*f*_ denote the mass ratio of rubber and filler in the un-swollen compounds.

Dynamic mechanical analysis (DMA) was performed in tension mode using a dynamic mechanical analyzer (TA Instruments DMA Q800, USA). Rectangular specimens with approximate dimensions of 6 × 30 × 1.2 mm were prepared and tested. All tests were carried out at a temperature range of − 100 °C to 80 °C at a fixed frequency of 1 Hz and a heating rate of 2 °C/min. The dynamic strain was set at 0.08%. Hardness measurement was conducted as per ISO 48-4 with a Shore A durometer (Wallace Instruments, model H17A, UK). Tensile test was performed according to ISO 37 (Die 1) using a universal testing machine (Instron, model 5567A, Norwood, Massachusetts, USA). The scanning electron microscope (SEM; Leo 1450VP, Cambridge, UK) was used to observe morphology of the tensile fracture surfaces of the filled ENR nanocomposites. The fracture surfaces were sputter-coated with gold prior to the microscopic observation.

### Ethics declarations

The collection and experimental research on Napier grass comply with the Plant Variety Protection Act, Thailand (1999).

## Supplementary Information

Supplementary Figures.

## Data Availability

The datasets generated and/or analysed during the current study are available at https://chem.sc.kku.ac.th/khatcharin/expt-data.
