# Supplementary material for: Enhanced interfacial interaction between modified cellulose nanocrystals and epoxidized natural rubber via ultraviolet irradiation
Source: Sci Rep. 2022 Apr 23;12:6682. doi: 10.1038/s41598-022-10558-5 (PMC9035154; doi:10.1038/s41598-022-10558-5)
Supplement: Supplementary file 1 — Supplementary Figures. [file 41598_2022_10558_MOESM1_ESM.pdf]

Supplementary Information

Enhanced Interfacial Interaction between Modified Cellulose Nanocrystals and Epoxidized Natural Rubber *via* Ultraviolet Irradiation

Oranooch Somseemee[1], Pongdhorn Saeoui[2], Florian T. Schevenels[3], and Chomsri Siriwong[1,3*]

[1]Materials Chemistry Research Center (MCRC-KKU), Department of Chemistry and Center of Excellence for Innovation in Chemistry (PERCH-CIC), Faculty of Science, Khon Kaen University, Khon Kaen 40002, Thailand

[2]National Metal and Materials Technology Center (MTEC), National Science and Technology Development Agency (NSTDA), 114 Thailand Science Park, Pathum Thani 12120, Thailand

[3]Department of Chemistry, Faculty of Science, Khon Kaen University, Khon Kaen 40002, Thailand

(*) Corresponding author: Chomsri Siriwong

E-mail address: schoms@kku.ac.th (C. Siriwong)

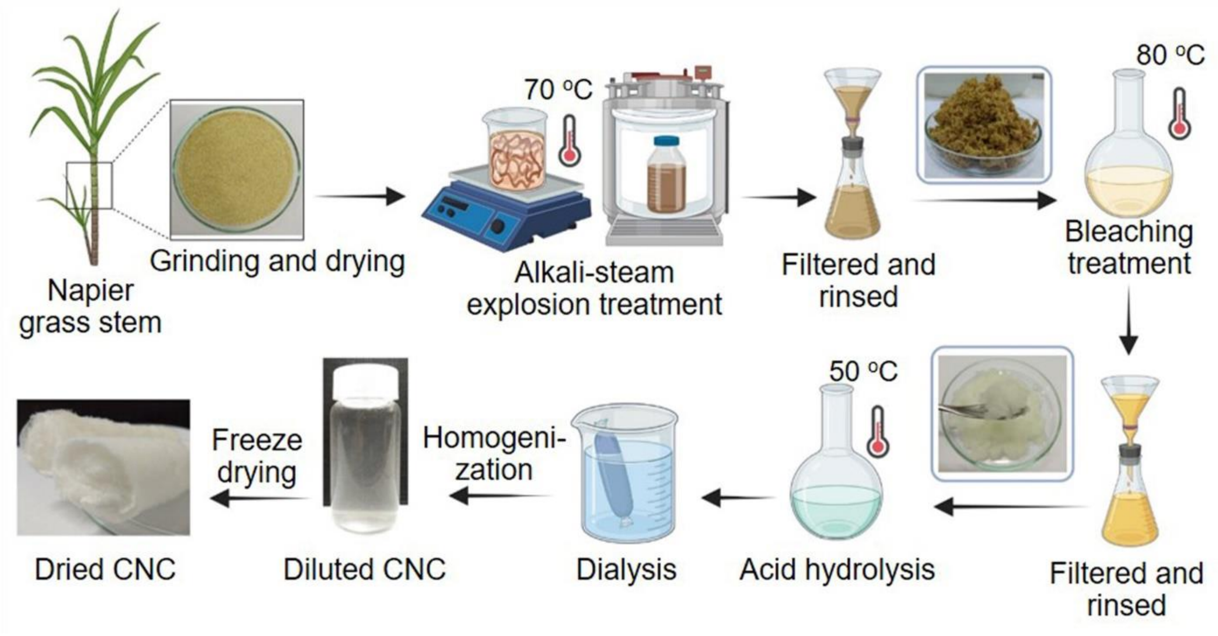

Figure S1. Diagram of the CNC extraction.

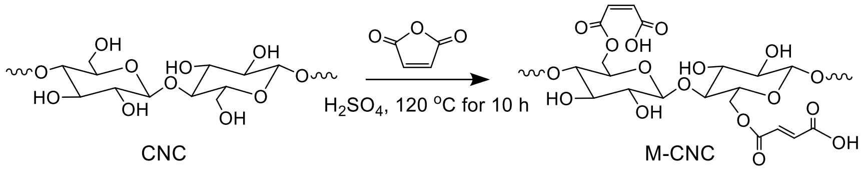

Figure S2 The chemical reaction of MAH modified CNCs surface.

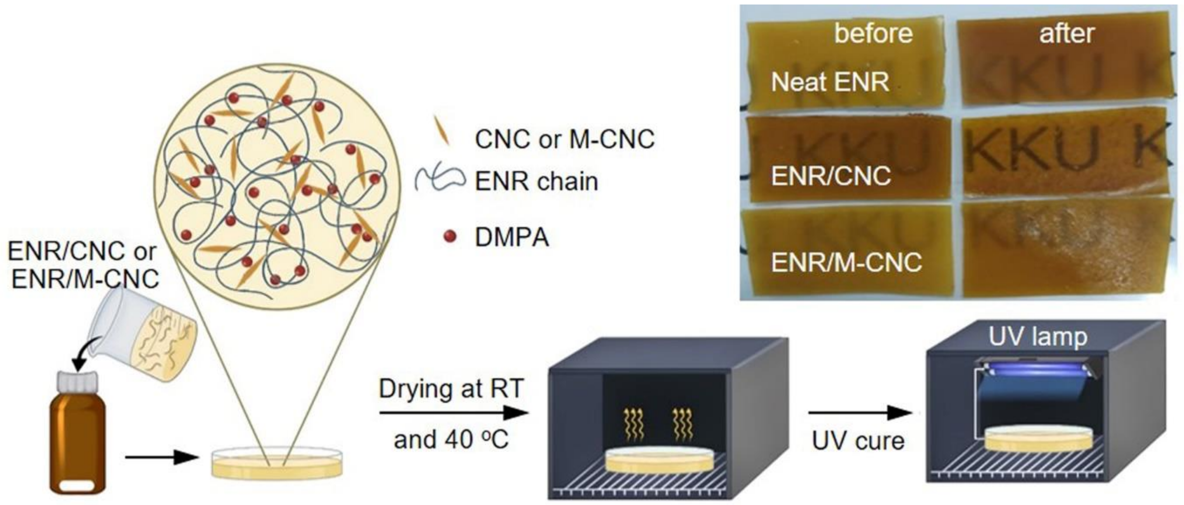

Figure S3. Schematic illustration of the UV-cured nanocomposite preparation.
